# Effect of a Community-Based Hepatitis B Virus Infection Detection Combined with Vaccination Program in China

**DOI:** 10.3390/vaccines10010019

**Published:** 2021-12-24

**Authors:** Xinyao Liu, Wuqi Qiu, Yan Liang, Wei Zhang, Qian Qiu, Xinxin Bai, Guolin Dai, Hao Ma, Hongpu Hu, Wei Zhao, Guangyu Hu

**Affiliations:** 1 Institute of Medical Information, Chinese Academy of Medical Sciences and Peking Union Medical College, Beijing 100020, China; liu.xinyao@imicams.ac.cn (X.L.); qiu.wuqi@imicams.ac.cn (W.Q.); dai.guolin@imicams.ac.cn (G.D.); ma_hao@imicams.ac.cn (H.M.); 2 Chaoyang District Center for Disease Prevention and Control of Beijing, Beijing 100021, China; cyqwjwcdcly@bjchy.gov.cn (Y.L.); 20202184@stu.hebmu.edu.cn (X.B.); 3 Beijing Center for Disease Prevention and Control, Beijing 100013, China; zhangweipumc@163.com (W.Z.); bjcdckjb@wjw.beijing.gov.cn (Q.Q.); 4 School of Public Health, Hebei Medical University, Shijiazhuang 050011, China; 5 Qinghai Center for Disease Prevention and Control, Xining 810007, China

**Keywords:** China, adult, hepatitis B virus, screening, vaccination

## Abstract

Evidence on the effectiveness of hepatitis B virus (HBV) infection screening and vaccination programs remains rare in China. We used a quasi-experimental method, propensity score matching, to evaluate the effects of a community-based HBV infection detection combined with vaccination (HBVIDV) program in a pilot. Data were retrieved from the HBVIDV program implemented between July 2019 and June 2020. Outcomes were the difference between the treatment and control groups in hepatitis B vaccination (≥1 dose), hepatitis B vaccine series completion (≥3 doses), and serologic evidence of vaccine-mediated immunity. Altogether, 26,180 individuals were included, where 6160 (23.5%) individuals were assigned to the treatment group, and 20,020 (76.5%) individuals were assigned to the control group. After propensity score matching, 5793 individuals were matched. The rates of hepatitis B vaccination, hepatitis B vaccine series completion, and prevalence of vaccine-mediated immunity in the treatment and control groups were 29.0% vs. 17.8%, 22.1% vs. 13.1%, and 38.2% vs. 27.6%, respectively. The HBVIDV program was significantly associated with increased hepatitis B vaccination rate (OR, 1.884, 95% CI 1.725–2.057), hepatitis B vaccine series completion rate (OR, 1.872, 95% CI 1.696–2.065), and prevalence of vaccine-mediated immunity (OR, 1.623, 95% CI 1.501–1.755). The greater magnitude of association between HBVIDV program and outcomes was observed among adults aged 35–54 years and adults who live in rural areas. The HBVIDV program was effective in increasing the hepatitis B vaccination rate, hepatitis B vaccine series completion rate, and prevalence of vaccine-mediated immunity among adults in the pilot. Further focusing the program on special populations and regions may produce more effective results.

## 1. Introduction

The World Health Assembly proposed the global health sector strategy (GHSS) to eliminate viral hepatitis as a public health threat by 2030, with a goal of reducing the incidence of chronic hepatitis infection by 90% and reducing the annual deaths owing to hepatitis by 65% [1]. China has the largest hepatitis B virus (HBV) infection burden, with approximately 86 million hepatitis B surface antigen (HBsAg) carriers in 2016, accounting for one-third of the world’s total cases [2]. Therefore, effective responses in China are essential to achieve the 2030 GHSS goals.

In the past 30 years, China has implemented a series of comprehensive prevention and control measures for hepatitis B, and significant progress has been made. The implementation of the hepatitis B vaccination program for newborn and infant, hepatitis B vaccine catch-up campaign for children aged <15 years, and comprehensive program to prevent mother-to-child transmission [3,4,5] has resulted in the >95% coverage of both children’s three doses and timely birth dose of hepatitis B vaccine [4,5], and the <1% prevalence of HBV infection among children aged <5 years [5,6]. China has gradually narrowed the gap with the 2030 GHSS target of reducing the incidence of chronic hepatitis infection by 90%; however, there are still some challenges in reducing mortality by 65%. Although China has required medical institutions to screen HBV infection since 2009 [7] and launched a national preconception health examination project in 2010 [5], only 19% and 10–11% of chronic hepatitis B infections are diagnosed and treated [5]. The World Health Organization (WHO) estimated that approximately 10 million people may die from cirrhosis and liver cancer (most of which are caused by hepatitis B) in China between 2015 and 2030 if effective measures are not undertaken [8].

It is recommended that all adults have routine access to and be offered HBsAg serological testing with linkage to prevention, care, and treatment services in setting with a ≥2% or ≥5% HBsAg seroprevalence in the general population [9]. In 2016, a modeling study in China reported that HBV infection screening and hepatitis B vaccination in adults aged 21–39 years can prevent 1.9 million HBV infections and 56,000 HBV-related early deaths [10]. Another modeling study in 2021 in China has revealed that HBV infection screening and hepatitis B vaccination for adults yield 18.08 life years and 17.46 quality-adjusted life years [11]. However, the evidence for the effectiveness of such public health projects funded by the government is insufficient.

The Chinese government has established national-level viral hepatitis prevention and control demonstration areas since 2008 [12]. As a demonstration area, Chaoyang District, Beijing implemented a community-based HBV infection detection combined with vaccination (HBVIDV) program from December 2013 to June 2015 (first wave) and from July 2019 to June 2020 (second wave), which provides free HBV infection detection and vaccination to permanent residents in 43 communities in the jurisdiction. Therefore, this study used hepatitis B vaccination (≥1 dose), hepatitis B vaccine series completion (≥3 doses), and vaccine-mediated immunity as measurement indicators to evaluate the early impact of the HBVIDV program in adults. We hypothesized that the HBVIDV program would significantly improve the status of adult hepatitis B vaccination.

## 2. Materials and Methods

### 2.1. Study Design

Using propensity score matching, we evaluated the effect of the HBVIDV program on outcomes among adults who were recruited or not recruited in this program. Data from the HBVIDV program implemented from July 2019 to June 2020 were used for analysis. This study was reported following the Strengthening the Reporting of Observational Studies in Epidemiology (STROBE) guideline [13].

### 2.2. Setting

Chaoyang District has the largest population in Beijing, China [14]. The HBsAg prevalence in adults aged ≥20 years in 2010 and 2015 was 2.97% and 2.99%, respectively [15,16]. In December 2013, Chaoyang District conducted a pilot study of a community-based HBVIDV program in four communities for permanent residents and officially launched the program in 39 other communities from May 2014 to June 2015. This was the first wave of implementation of the HBVIDV program, and the second wave of implementation was conducted from July 2019 to June 2020. The specific process of program implementation was as follows: first, trained residents’ committee staff posted the notice of investigation and physical examination in the area in advance. Then, the program staff conducted questionnaire surveys among residents who volunteered to participate through surveys in concentrated places or at home. Blood samples were collected by the staff of the community health service center in the community sites selected by the community neighborhood committee. After obtaining 5 mL of venous blood from each participant, each community health service center centrifuged the blood collected, stored it at 4 °C, and transported it to the designated laboratory on that day. The serological test used the enzyme-linked immunosorbent assay kits of Beijing Wantai Biotechnology Company. Finally, the serological results were fed back to the participants within two months by the person in charge of each community, and the “Hepatitis B Vaccine Free Vaccination Notice” was issued to those who were seronegative for HBsAg and anti-hepatitis B surface (anti-HBs) and recommended that participants go to the corresponding community health service center for vaccination.

### 2.3. Study Population

Respondents in the HBVIDV program were recruited from 43 communities, and the respondents must be permanent residents of Chaoyang District. Permanent residents refer to those who lived within the Chaoyang District for more than six months throughout the past year. The HBVIDV program was approved by Beijing Center for Disease Prevention and Control Ethical Committee. Written informed consent was obtained from all participants.

**Figure 1:**
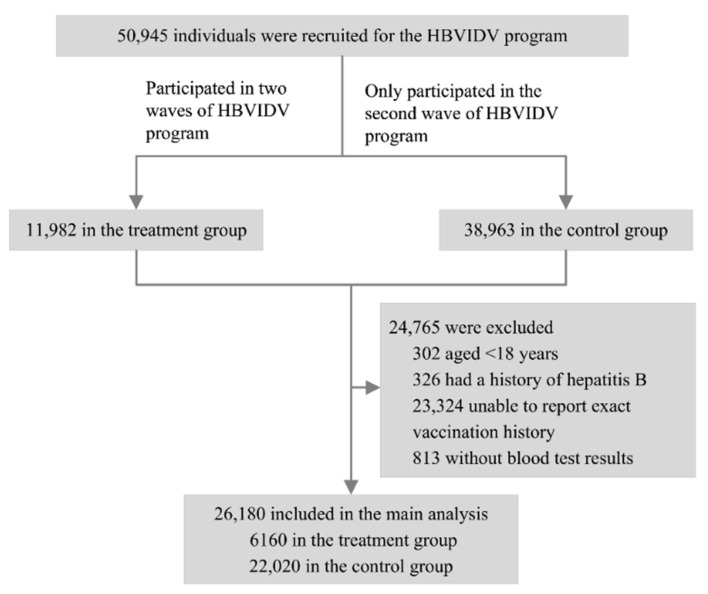
Flowchart of study participant selection for HBVIDV program from July 2019 to June 2020.

Altogether, 109,764 and 50,945 individuals were recruited in a two-wave survey, respectively. All participants were assigned a unique identification code when they first participated in the HBVIDV program. According to the code, individuals were assigned to the treatment group if they were indexed to participate in two-wave surveys. Those who only participated in the second wave of surveys but did not participate in the first wave of surveys were assigned to the control group. Individuals who were aged <18 years, had previous hepatitis B, did not report exact vaccination history, or were without blood test results were excluded. The selection process of individuals included in the study is shown in Figure 1.

### 2.4. Measurements

#### 2.4.1. Covariates

Individuals reported their sociodemographic information and health condition through questionnaire surveys. The participants were categorized according to age as follows: 18–34, 35–44, 45–54, 55–64, and ≥65 years. Education level was categorized into “illiterate”, “primary school/junior high school”, “senior high school”, and “college graduate or above”. Employment condition was categorized as “working”, “retired”, “unemployed”, and “student”. Economic level was categorized as “far below average”, “below average”, “average”, “above average”, and “far above average” by asking, “What is the economic level of your family in local?”. Self-rated socioeconomic status was categorized into “higher”, “equality”, and “lower” by asking, “Compared to peers, what do you think of your socioeconomic status?”. Multimorbidity was defined as a person suffering from two or more chronic diseases or conditions [17]. By asking “Were you diagnosed with the following diseases by a community health service center or above-level medical institution”, the prevalence of the following 11 self-reported diseases was identified: hypertension; diabetes; dyslipidemia; chronic obstructive pulmonary disease; stroke; heart disease; asthma; bone and joint disease; digestive system disease; urinary system disease; and cancer or malignant tumor. Self-rated health condition was categorized as “very unhealthy”, “unhealthy”, “fair healthy”, “healthy”, and “very healthy”.

#### 2.4.2. Outcome Definition

The primary outcome was hepatitis B vaccination (≥1 dose). By asking “Have you ever been hepatitis B-vaccinated before?”, the history of hepatitis B vaccination was identified. The secondary outcome included hepatitis B vaccine series completion (≥3 doses) and serological evidence of vaccine-mediated immunity. Hepatitis B vaccine series completion was identified using the following question: if the above answer is “Yes”, “How many doses of hepatitis B vaccine have you received?”. Serologic evidence of vaccine-mediated immunity was required, which was defined as seronegativity for hepatitis B core antibody and seropositivity for anti-HBs [18].

### 2.5. Statistical Analysis

Distributions of characteristics are presented using frequencies (%) for categorical variables. Propensity score matching was used to evaluate the association between the HBVIDV program and outcomes. The propensity score of an individual is the probability that an individual chooses to participate in the HBVIDV program given a set of covariate characteristics [19]. The propensity score was calculated using the method of multivariate logistic regression, and the following covariates were included in the multivariate logistic regression model as factors affecting an individual who participated in the HBVIDV program: age, gender, education level, employment condition, economic level, self-rated socioeconomic status, living area, multimorbidity, and self-rated health condition. The missing data of categorical covariates were coded as the “Missing” category and were included in the propensity score matching analysis [20]. After the propensity score was estimated, a 1:1 match without replacement was performed using nearest neighbor matching with a 0.2 SD caliper width. The standard mean difference (SMD) was within 0.1, which was considered to be the balance between the treatment and control groups. Once a matched data set was obtained, the association between the HBVIDV program and outcomes was estimated using odds ratios (ORs) and 95% confidence intervals (CIs) calculated from logistic regression.

**Figure 2:**
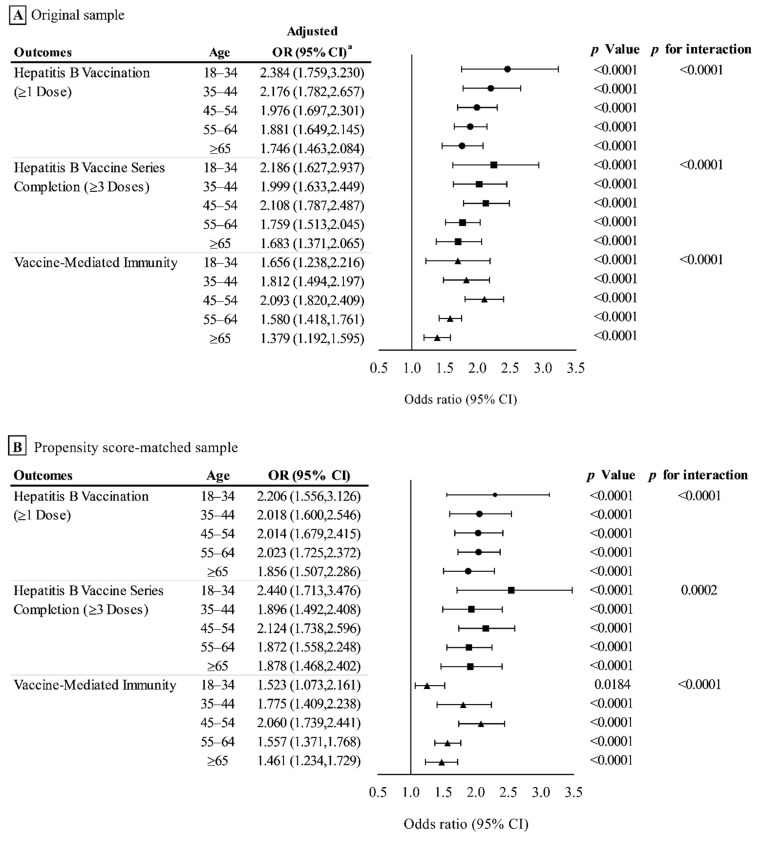

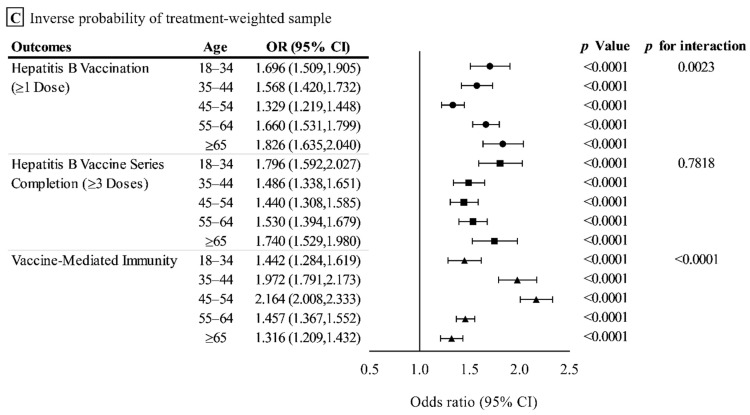
Association between HBVIDV program and outcomes in original sample (**A**), propensity score–matched sample (**B**) and inverse probability of treatment–weighted sample (**C**), stratified by age. ^a^ Odds ratios were adjusted for gender, educational level, employment condition, economic level, self-rated socioeconomic status, living area, multimorbidity, self-rated health condition.

**Figure 3:**
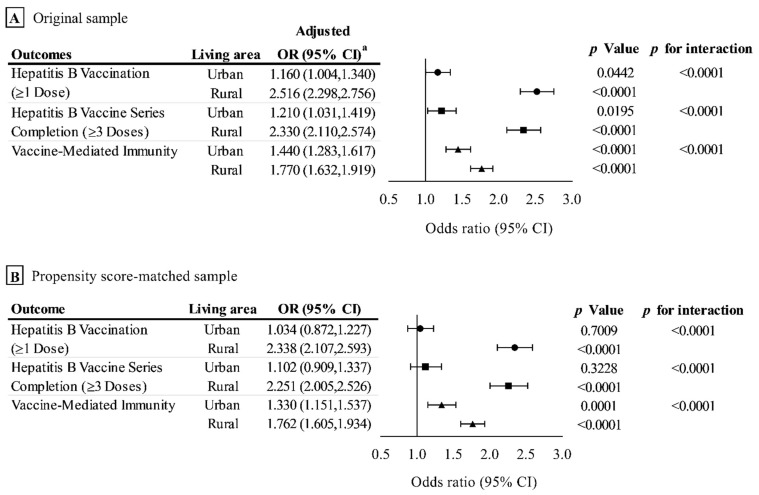

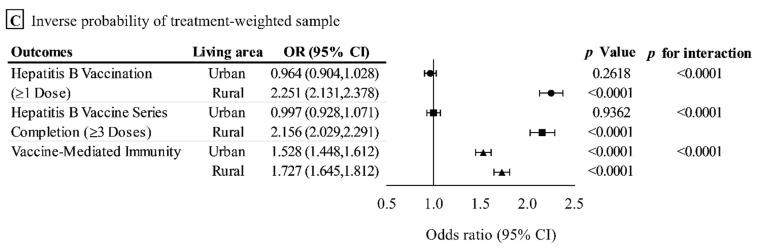
Association between HBVIDV program and outcomes in original sample (**A**), propensity score–matched sample (**B**) and inverse probability of treatment–weighted sample (**C**), stratified by living area. ^a^ Odds ratios were adjusted for age, gender, educational level, employment condition, economic level, self-rated socioeconomic status, multimorbidity, self-rated health condition.

Additionally, we performed subgroup analyses by age and living area (results are shown in Figure 2 and Figure 3; complete logistic regression results were shown in Supplemental File S1).

We conducted sensitivity analyses to ensure the robustness of the research results. First, we used inverse probability of treatment weighting (IPTW) to evaluate the association between the HBVIDV program and outcomes. IPTW is a form of propensity score analysis that uses mutual weighting of propensity scores to balance the covariate characteristics between the treatment and control group [19]. The IPTW model was created from original samples and weights based on propensity scores (results in Table 1 and Table 2, Figure 2C and Figure 3C). Secondly, we analyzed the relationship between the HBVIDV program and serological evidence of vaccine-mediated immunity after only excluding those younger than 18 years, those with previous history of hepatitis B, and those with no serological examination results. In the main analysis, we excluded individuals with missing outcome data. However, of the 50,945 individuals included in the second wave of HBVIDV, only 2320 (4.6%) individuals lack serological results. Therefore, in order to explain the accuracy of the study results, we analyzed the association between the HBVIDV program and serological evidence of vaccine-mediated immunity after only excluding 2882 (6%) individuals according to the above criteria (results in Supplemental File S2). In this part, the missing data of covariates were still classified as “Missing” and were included in the propensity score matching and IPTW models. Finally, we analyzed the association between the HBVIDV program and serological evidence of vaccine-mediated immunity in the complete sample set, that is, individuals who were younger than 18 years old, had past hepatitis B history, and were missing serological tests and covariates were excluded. In the above analysis, missing values of covariates were coded as categorical variables. Therefore, we analyzed the relationship between the HBVIDV program and serological evidence of vaccine-mediated immunity in a complete sample set to determine the robustness of research results (results in Supplemental File S3).

All *p* values were two-sided, and *p* < 0.05 was considered significant. All statistical analyses were conducted using R version 4.0.5 and SAS version 9.4.

## 3. Results

### 3.1. Participant Characteristics

After applying the exclusion criteria, a total of 26,180 individuals were included in main study. Altogether, 6160 (23.5%) individuals who participated in two waves of HBVIDV program were assigned to the treatment group, and 22,020 (76.5%) individuals who only participated in the second wave were assigned to the control group. Before matching, the characteristics of the treatment and control groups were unbalanced; the treatment group was older, less educated, had the most retired individuals, had more individuals living in rural areas, and had more individuals with multimorbidity (Table 1).

The propensity score matching resulted in 5793 individuals in the treatment group and 5793 individuals in the control group who were matched, and most of the characteristics of the two groups were well balanced (SMD < 0.1) (Table 1).

### 3.2. Outcome Analysis in the Original Sample

In the original sample, the hepatitis B vaccination rate, hepatitis B vaccine series completion rate, and the prevalence of vaccine-mediated immunity in the treatment and control groups were 27.7% vs. 18.9%, 21.1% vs. 14.2%, and 38.0% vs. 31.0%, respectively. The associations between the HBVIDV program and outcomes in original sample are shown in Table 2. The results of subgroup analysis are shown in Figure 2A and Figure 3A. Results of complete subgroup analysis are shown in Supplemental File S1.

### 3.3. Outcome Analysis in the Propensity Score-Matched Sample

#### 3.3.1. Effect on Hepatitis B Vaccination (≥1 Dose)

After propensity score matching, the hepatitis B vaccination rate in the treatment and control groups was 29.0% and 17.8%, respectively. The HBVIDV program was significantly associated with an increased hepatitis B vaccination rate, with an OR of 1.884 (95% CI, 1.725–2.057) (Table 2). In the subgroup analysis, a greater magnitude of association between HBVIDV program and hepatitis B vaccination was observed among adults aged 18–34 years (Figure 2B), and adults who live in rural areas (Figure 3B), with ORs of 2.206 (95% CI, 1.556–3.126) and 2.338 (95% CI, 2.107–2.593), respectively (*p* for interaction < 0.0001).

#### 3.3.2. Effect on the Hepatitis B Vaccine Series Completion (≥3 Doses)

For hepatitis B vaccine series completion rate, the treatment and control groups were 22.1% and 13.1%, respectively. The HBVIDV program was significantly associated with an increased hepatitis B vaccine series completion rate, with an OR of 1.872 (95% CI, 1.696–2.065) (Table 2). As with the above results, a greater magnitude of association between the HBVIDV program and hepatitis B vaccine series completion was observed among adults aged 18–34 years, with an OR of 2.440 (95% CI, 1.713–3.476) (*p* for interaction *=* 0.0002) (Figure 2B), and adults who live in rural areas, with an OR 2.251 (95% CI 2.005–2.526) (*p* for interaction < 0.0001) (Figure 3B).

#### 3.3.3. Effect on the Prevalence of Vaccine-Mediated Immunity

Following propensity score matching, the prevalence of vaccine-mediated immunity in the treatment and control groups was 38.2% and 27.6%, respectively. The increased prevalence of vaccine-mediated immunity was significantly associated with the HBVIDV program, with an OR of 1.623 (95% CI, 1.501–1.755) (Table 2). In the subgroup analysis, the largest magnitude of association was observed among adults aged 35–44 and 45–54, with ORs of 1.775 (95% CI 1.409–2.238) and 2.060 (95% CI 1.739–2.441), respectively (*p* for interaction <0.0001) (Figure 2B); and adults who live in rural areas, with an OR of 1.762 (95% CI 1.605–1.934) (*p* for interaction <0.0001) (Figure 3B).

### 3.4. Sensitivity Analysis

The results of sensitivity analysis with IPTW were similar to those of propensity score matching and are shown in Table 1 and Table 2 and Figure 2C and Figure 3C. The sensitivity analysis in HBVIDV program and vaccine-mediated immunity were largely unchanged and are shown in Supplemental Files S2 and S3.

## 4. Discussion

This study provides recommendations for the formulation of HBV infection screening and vaccination policies in areas where the prevalence of HBsAg is >2%. Overall, this study found that, compared with those who were not recruited in the HBVIDV program, those who were recruited had a higher hepatitis B vaccination rate, hepatitis B vaccine series completion rate, and prevalence of vaccine-mediated immunity. After propensity score matching, the hepatitis B vaccination rate, hepatitis B vaccine series completion rate, and the prevalence of vaccine-mediated immunity in the treatment and control groups were 29.0% vs. 17.8%, 22.1% vs. 13.1%, and 38.2% vs. 27.6%, respectively. Moreover, the HBVIDV program has a more significant effect on adults aged 35–54 years and live in rural areas.

Previous studies from several countries have reported that HBV infection screening combined with hepatitis B vaccination, care, or treatment increased the detection rate of hepatitis B [21,22,23,24], vaccination coverage rate [21,25,26], medical care rate [27,28,29,30], and treatment compliance [31]. However, these studies did not evaluate the effectiveness of the program implementation in comparison with the national conventional model, that is, compared with the absence of these measures. This study found that, compared with the conventional model, the implementation of the HBVIDV program in China has effectively increased the adult hepatitis B vaccination rate, hepatitis B vaccine series completion rate, and the prevalence of vaccine-mediated immunity. Moreover, the hepatitis B vaccination rate and antibody levels of Chinese adults recruited to the HBVIDV program were higher than the levels reported in previous studies. Previous studies reported that the hepatitis B vaccine rate and antibody level of Chinese adults have been at a low level, the hepatitis B vaccination rate ranged from 17.7% to 31.2% between 2009 and 2019 [25,26,32], and the prevalence of vaccine-mediated immunity ranged from 14.2% to 34.0% between 2010 and 2014 [33,34,35,36].

In this study, the largest difference in vaccine-mediated immunity between the treatment group and the control group was in the aged 35–44 and 45–54 years, and the difference in hepatitis B vaccination rate and series completion rate was also greater, which indicates that the HBVIDV program was more effective for age group 35–54 years. However, at the same time, this also revealed that these populations are at a high risk of HBV infection. Recent studies reported that HBV infection has shown a significant increasing trend with age [37,38,39]. A study reported that the median age of the population with CHB increased from 48 years in 2006 to 52 years in 2015 [40,41]. Thus, conducting hepatitis B screening and vaccination for the age group 35–54 years is crucial. Although we observed the largest difference in hepatitis B vaccination and series completion rates between the treatment group and the control group was in the 18–34 age group, it is difficult to determine whether this is due to the HBVIDV program, because these groups may benefit from a number of measures launched by China since 1992, such as the newborn and infant hepatitis B vaccination program, the childhood hepatitis B vaccine catch-up campaign, and the comprehensive program to prevent mother-to-child transmission [4,5,7]. We observed that the difference in the outcomes between the treatment group and the control group among older adults was small; this may be due to the decline in the hepatitis B vaccination rate due to the increase in age. This result is similar to those of other studies [18,35].

Additionally, the HBVIDV program appeared more effective in rural areas than in urban areas. This is an important finding regarding the significance of public health. The prevalence of HBV infection in rural China has been reported to be higher than that in urban areas [42,43]. Moreover, the hepatitis B vaccine coverage rate and the prevalence of vaccine-mediated immunity in rural areas are also low [35,36,44,45].

The modeling studies of China showed that launching an extensive adult HBV infection screening and vaccination program can effectively reduce incidence and mortality, and it is cost-effective [10,11]. Our research provides real-world evidence for this. Overall, this study supports an HBV infection screening and vaccination campaign for adults, which will considerably improve the status of adult hepatitis B vaccination and reduce incidence and mortality. In addition, we also found the HBVIDV program actively mobilized the populations of low education level and rural area to participate in HBV infection screening and vaccination. These populations are the main reasons for the low rate of adult hepatitis B vaccination [26,44,46]. The HBVIDV program has also significantly mobilized non-multimorbidity and self-rated health population to participate in screening and vaccination. China’s free hepatitis B screening is only for unpaid blood donors and preconception health examination [5], and adult hepatitis B vaccination has not been included in the immunization program. Therefore, it is also important to detect the status of HBV infection and vaccinate in the general population. In short, when the country has not made a decision on the large-scale implementation of adult hepatitis B screening and vaccination policies, this study provides positive evidence for the government to fund this project to bridge the gap with the 2030 GHSS targets. However, it is necessary to further evaluate affordability and feasibility from the perspective of the government.

However, our study has some limitations. First, this was a cross-sectional survey. The control group lacked the baseline hepatitis B vaccination rate and could not be compared longitudinally. However, we compared hepatitis B vaccination rates and prevalence of vaccine-mediated immunity with previous studies of China. Second, hepatitis B vaccination was self-reported by individuals, resulting in a low reported hepatitis B vaccination rate. However, this study also reported the vaccine-mediated immunity level of serological evidence, which can also reflect the population’s vaccination level. However, it should be noted that the production of antibodies after vaccination of hepatitis B vaccine is easily affected by many factors, such as age, diabetes, tumors, and other diseases, autoimmune function, and vaccination from different manufacturers. Therefore, there may still be deviations between the vaccination rate reported in this study and the actual vaccination rate. Lastly, although we adjusted for a series of factors through propensity score matching and IPTW, there may still be potential confounding factors.

## 5. Conclusions

The government-funded HBVIDV program in China effectively increased the hepatitis B vaccination rate, hepatitis B vaccine series completion rate, and the prevalence of vaccine-mediated immunity in adults. The potential benefit of the pilot program would have been expanded in the labor population aged 35–54 years and residents in rural areas in future.

## Figures and Tables

**Table 1:** Characteristic of total sample, original sample, propensity score-matched sample, and inverse probability of treatment-weighted sample.

Characteristic	Total Sample (N = 26,180)	Original Sample			Propensity 1:1 Matching			IPTW Sample		
		Treatment Group (N = 6160)	Control Group (N = 20,020)	SMD	Treatment Group (N = 5793)	Control Group (N = 5793)	SMD	Treatment Group (N = 6160)	Control Group (N = 20,020)	SMD
Age				0.348			0.068			0.026
18–34	2625 (10.0)	248 (4.0)	2377 (11.9)		248 (4.3)	283 (4.9)		2062.4 (7.5)	2682.9 (10.4)	
35–44	2992 (11.4)	604 (9.8)	2388 (11.9)		604 (10.4)	587 (10.1)		4354.8 (15.9)	2848.1 (11.0)	
45–54	5826 (22.3)	1241 (20.2)	4585 (22.9)		1241 (21.4)	1323 (22.8)		7043.7 (25.7)	5811.3 (22.5)	
55–64	9616 (36.7)	2400 (39.0)	7216 (36.0)		2222 (38.4)	2397 (41.4)		8485.0 (31.0)	9799.8 (37.9)	
≥65	5121 (19.6)	1667 (27.1)	3454 (17.3)		1478 (25.5)	1203 (20.8)		5431.0 (19.8)	4745.4 (18.3)	
Gender, male	9593 (36.6)	2075 (33.7)	7518 (37.6)	0.081	1965 (33.9)	1868 (32.3)	0.036	9812.8 (35.8)	9471.5 (36.6)	0.015
Education level				0.255			0.052			0.085
Illiterate	514 (2.0)	135 (2.2)	379 (1.9)		135 (2.3)	117 (2.0)		476.1 (1.7)	526.0 (2.0)	
Primary school/Junior high school	8768 (33.5)	2598 (42.2)	6170 (30.8)		2598 (44.9)	2616 (45.2)		8757.8 (32.0)	8727.4 (33.7)	
Senior high school	5769 (22.0)	1330 (21.6)	4439 (22.2)		1330 (23.0)	1431 (24.7)		5541.4 (20.2)	5913.9 (22.8)	
College graduate or above	4114 (15.7)	899 (14.6)	3215 (16.1)		899 (15.5)	919 (15.9)		4620.7 (16.9)	4082.2 (15.8)	
Missing	7015 (26.8)	1198 (19.5)	5817 (29.1)		831 (14.3)	710 (12.3)		7981.0 (29.2)	6638.0 (25.6)	
Employment condition				0.388			0.030			0.082
Working	9025 (34.5)	1803 (29.3)	7222 (36.1)		1803 (31.1)	2138 (36.9)		6850.9 (25.0)	9614.7 (37.1)	
Retired	9017 (34.4)	3160 (51.3)	5857 (29.3)		2793 (48.2)	2572 (44.4)		10,373.2 (37.9)	8240.2 (31.8)	
Unemployed	1582 (6.0)	487 (7.9)	1095 (5.5)		487 (8.4)	371 (6.4)		2720.6 (9.9)	1363.3 (5.3)	
Student	33 (0.1)	4 (0.1)	29 (0.1)		4 (0.1)	2 (0.0)		8.6 (0.0)	31.3 (0.1)	
Missing	6523 (24.9)	706 (11.5)	5817 (29.1)		706 (12.2)	710 (12.3)		7423.6 (27.1)	6638.0 (25.6)	
Economic level				0.229			0.062			0.077
Far below average	1107 (4.2)	277 (4.5)	830 (4.2)		277 (4.8)	308 (5.3)		1049.5 (3.8)	1170.0 (4.5)	
Below average	3719 (14.2)	1090 (17.7)	2629 (13.1)		1090 (18.8)	1065 (18.4)		4171.4 (15.2)	3641.8 (14.1)	
Average	13,805 (52.7)	3467 (56.3)	10,338 (51.6)		3467 (59.9)	3593 (62.2)		13,609.2 (49.7)	13,910.4 (53.7)	
Above average	468 (1.8)	108 (1.8)	360 (1.8)		108 (1.9)	105 (1.8)		469.5 (1.7)	468.1 (1.8)	
Far above average	61 (0.2)	15 (0.2)	46 (0.2)		15 (0.3)	12 (0.2)		50.9 (0.2)	59.1 (0.2)	
Missing	7020 (26.8)	1203 (19.5)	5817 (29.1)		836 (14.4)	710 (12.3)		8026.4 (29.3)	6638.0 (25.6)	
Self-rated socioeconomic status				0.217			0.065			0.082
Higher	584 (2.2)	154 (2.5)	430 (2.2)		154 (2.7)	127 (2.2)		767.5 (2.8)	558.5 (2.2)	
Equality	14,914 (57.0)	3784 (61.4)	11,130 (55.6)		3784 (65.3)	3970 (68.5)		14,658.0 (53.5)	15,027.8 (58.1)	
Lower	3662 (14.0)	1019 (16.5)	2643 (13.2)		1019 (17.6)	986 (17.0)		3925.0 (14.3)	3663.2 (14.2)	
Missing	7020 (26.8)	1203 (19.5)	5817 (29.1)		836 (14.4)	710 (12.3)		8026.4 (29.3)	6638.0 (25.6)	
Living area				0.433			0.002			0.020
Urban	11,149 (42.6)	1660 (27.0)	9489 (47.4)		1660 (28.7)	1654 (28.6)		12,029.2 (43.9)	11,124.0 (43.0)	
Rural	15,031 (57.4)	4500 (73.1)	10531 (52.6)		4133 (71.3)	4139 (71.5)		15,347.6 (56.1)	14,763.5 (57.0)	
Multimorbidity							0.065			0.084
Yes	4654 (17.8)	1639 (26.6)	3015 (15.1)	0.198	1639 (28.3)	1655 (28.6)		5011.4 (18.3)	4738.4 (18.3)	
No	14,506 (55.4)	3318 (53.4)	11,188 (55.9)		3318 (57.3)	3428 (59.2)		14,339.1 (52.4)	14,511.0 (56.1)	
Missing	7020 (26.8)	1203 (19.5)	5817 (29.1)		836 (14.4)	710 (12.3)		8026.4 (29.3)	6638.0 (25.6)	
Self-rated health condition				0.250			0.063			0.072
Very unhealthy	496 (1.9)	136 (2.2)	360 (1.8)		136 (2.4)	132 (2.3)		461.8 (1.7)	514.7 (2.0)	
Unhealthy	1405 (5.4)	424 (6.9)	981 (4.9)		424 (7.3)	420 (7.3)		1539.8 (5.6)	1373.2 (5.3)	
Fair healthy	5995 (22.9)	1771 (28.8)	4224 (21.1)		1771 (30.6)	1860 (32.1)		6533.3 (23.9)	6013.6 (23.2)	
Healthy	8995 (34.4)	2117 (34.4)	6878 (34.4)		2117 (36.5)	2209 (38.1)		8391.2 (30.7)	9154.8 (35.4)	
Very healthy	2269 (8.7)	509 (8.3)	1760 (8.8)		509 (8.8)	462 (8.0)		2424.3 (8.9)	2193.2 (8.5)	
Missing	7020 (26.8)	1203 (19.5)	5817 (29.1)		836 (14.4)	710 (12.3)		8026.4 (29.3)	6638.0 (25.6)	

Abbreviations: SMD, standard mean difference; IPTW, inverse probability of treatment weighting; Categorical variables are reported as number (%).

**Table 2:** Association between HBVIDV program and outcomes.

	Hepatitis B Vaccination (≥1 Dose)				Hepatitis B Vaccine Series Completion (≥3 Doses)				Vaccine-Mediated Immunity			
	Treatment Group	Control Group	OR (95% CI)	*p* Value	Treatment Group	Control Group	OR (95% CI)	*p* Value	Treatment Group	Control Group	OR (95% CI)	*p* Value
Original sample unadjusted model	1708 (27.7)	3779 (18.9)	1.649 (1.543, 1.761)	<0.0001	1300 (21.1)	2839 (14.2)	1.619 (1.505, 1.741)	<0.0001	2339 (38.0)	6210 (31.0)	1.361 (1.283, 1.445)	<0.0001
Original sample adjusted model ^a^	1708 (27.7)	3779 (18.9)	1.958 (1.816, 2.111)	<0.0001	1300 (21.1)	2839 (14.2)	1.912 (1.761, 2.077)	<0.0001	2339 (38.0)	6210 (31.0)	1.672 (1.565, 1.785)	<0.0001
Propensity 1:1 Matching	1681 (29.0)	1033 (17.8)	1.884 (1.725, 2.057)	<0.0001	1278 (22.1)	761 (13.1)	1.872 (1.696, 2.065)	<0.0001	2211 (38.2)	1596 (27.6)	1.623 (1.501, 1.755)	<0.0001
IPTW sample	7227.7 (26.4)	4781.6 (18.5)	1.583 (1.519, 1.650)	<0.0001	5515.2 (20.2)	3580.4 (13.8)	1.572 (1.501, 1.646)	<0.0001	11328.6 (41.4)	7801.6 (30.1)	1.636 (1.539, 1.696)	<0.0001

Abbreviations: OR (95% CI), odds ratio (95% confidence interval). IPTW, inverse probability of treatment weighting. Outcome events (%) are reported. ^a^ Model was adjusted for age, gender, educational level, employment condition, economic level, self-rated socioeconomic status, living area, multimorbidity, self-rated health condition.

## Data Availability

The data presented in this study are available on request from the corresponding author. The data are not publicly available due to privacy.

## Supplementary Materials

The following supporting information can be downloaded at: https://www.mdpi.com/article/10.3390/vaccines10010019/s1, File S1. Complete Logistic Regression Results for Subgroup Analysis. File S2. Sensitivity Analysis of the Association Between HBVIDV Program and Serological Evidence of Vaccine-Mediated Immunity. File S3. Sensitivity Analysis of the Association between HBVIDV Program and Serological Evidence of Vaccine-Mediated Immunity in Complete Data Set.
