# Supplementary material for: Effect of a Community-Based Hepatitis B Virus Infection Detection Combined with Vaccination Program in China
Source: Vaccines (Basel). 2021 Dec 24;10(1):19. doi: 10.3390/vaccines10010019 (PMC8777927; doi:10.3390/vaccines10010019)
Supplement: Supplementary file 1 [file vaccines-10-00019-s001.zip › File S1.pdf]

File S1. Complete logistic regression results for subgroup analysis

Table S1. Association between HBVIDV program and outcomes in original sample, stratified by age

Table S2. Association between HBVIDV program and outcomes in propensity score-matched sample, stratified by age

Table S3. Association between HBVIDV program and outcomes in inverse probability of treatment-weighted sample, stratified by age

Table S4. Association between HBVIDV program and outcomes in original sample, stratified by living area

Table S5. Association between HBVIDV program and outcomes in propensity score-matched sample, stratified by living area

Table S6. Association between HBVIDV program and outcomes in inverse probability of treatment-weighted sample, stratified by living area

Table S1. Association between HBVIDV program and outcomes in original sample, stratified by age

Outcomes	Age	Treatment group (n=6160)	Control group (n=200020)	Unadjusted OR (95% CI)	Adjusted OR (95% CI) [a]	*p* Value
Hepatitis B Vaccination (≥1 Dose)	18–34	156 (62.9)	867 (36.5)	2.953 (2.252,3.871)	2.384 (1.759,3.230)	<0.0001
	35–44	323 (53.5)	747 (31.3)	2.525 (2.105,3.028)	2.176 (1.782,2.657)	<0.0001
	45–54	399 (32.2)	843 (18.4)	2.103 (1.828,2.421)	1.976 (1.697,2.301)	<0.0001
	55–64	491 (20.5)	915 (12.7)	1.771 (1.569,1.999)	1.881 (1.649,2.145)	<0.0001
	≥65	339 (20.3)	407 (11.8)	1.911 (1.632,2.238)	1.746 (1.463,2.084)	<0.0001
Hepatitis B Vaccine Series Completion (≥3 Doses)	18–34	131 (52.8)	671 (28.2)	2.847 (2.184,3.710)	2.186 (1.627,2.937)	<0.0001
	35–44	267 (44.2)	599 (25.1)	2.367 (1.966,2.849)	1.999 (1.633,2.449)	<0.0001
	45–54	320 (25.8)	616 (13.4)	2.239 (1.921,2.609)	2.108 (1.787,2.487)	<0.0001
	55–64	344 (14.3)	668 (9.3)	1.640 (1.427,1.885)	1.759 (1.513,2.045)	<0.0001
	≥65	238 (14.3)	285 (8.3)	1.852 (1.512,2.224)	1.683 (1.371,2.065)	<0.0001
Vaccine-Mediated Immunity	18–34	160 (64.5)	1196 (50.3)	1.795 (1.367,2.357)	1.656 (1.238,2.216)	<0.0001
	35–44	307 (50.8)	885 (37.1)	1.755 (1.467,2.101)	1.812 (1.494,2.197)	<0.0001
	45–54	491 (39.6)	1221 (26.6)	1.804 (1.582,2.057)	2.093 (1.820,2.409)	<0.0001
	55–64	825 (34.4)	1909 (26.5)	1.456 (1.319,1.608)	1.580 (1.418,1.761)	<0.0001
	≥65	556 (33.4)	999 (28.9)	1.230 (1.085,1.394)	1.379 (1.192,1.595)	<0.0001

Outcome evets (%) are reported.

[a]Model was adjusted for gender, educational level, employment condition, economic level, self-rated socioeconomic status, living area, multimorbidity, self-rated health condition.

Table S2. Association between HBVIDV program and outcomes in propensity score-matched sample, stratified by age

Outcomes	Age	Treatment group (n=5793)	Control group (n=5793)	OR (95% CI)	*p* Value
Hepatitis B Vaccination (≥1 Dose)	18–34	156 (62.9)	123 (43.5)	2.206 (1.556,3.126)	<.0001
	35–44	323 (53.5)	213 (36.3)	2.018 (1.600,2.546)	<.0001
	45–54	399 (32.2)	252 (19.1)	2.014 (1.679,2.415)	<.0001
	55–64	481 (21.7)	288 (12.0)	2.023 (1.725,2.372)	<.0001
	≥65	322 (21.8)	157 (13.1)	1.856 (1.507,2.286)	<.0001
Hepatitis B Vaccine Series Completion (≥3 Doses)	18–34	131 (52.8)	89 (31.5)	2.440 (1.713,3.476)	<.0001
	35–44	267 (44.2)	173 (29.5)	1.896 (1.492,2.408)	<.0001
	45–54	320 (25.8)	186 (14.1)	2.124 (1.738,2.596)	<.0001
	55–64	337 (15.2)	209 (8.7)	1.872 (1.558,2.248)	<.0001
	≥65	223 (15.1)	104 (8.7)	1.878 (1.468,2.402)	<.0001
Vaccine-Mediated Immunity	18–34	160 (64.5)	154 (54.4)	1.523 (1.073,2.161)	0.0184
	35–44	307 (50.8)	216 (36.8)	1.775 (1.409,2.238)	<.0001
	45–54	491 (39.6)	319 (24.1)	2.060 (1.739,2.441)	<.0001
	55–64	760 (34.2)	600 (25.0)	1.557 (1.371,1.768)	<.0001
	≥65	493 (33.4)	307 (25.5)	1.461 (1.234,1.729)	<.0001

Outcome evets (%) are reported.

Table S3. Association between HBVIDV program and outcomes in inverse probability of treatment-weighted sample, stratified by age

**Outcomes**	**Age**	**Treatment group (n=6160)**	**Control group (n=20020)**	**OR (95% CI)**	***p* Value**
Hepatitis B Vaccination (≥1 Dose)	18–34	1030.0 (49.9)	993.9 (37.0)	1.696 (1.509,1.905)	<0.0001
	35–44	1850.4 (42.5)	912.1 (32.0)	1.568 (1.420,1.732)	<0.0001
	45–54	1659.3 (23.6)	1094.1 (18.8)	1.329 (1.219,1.448)	<0.0001
	55–64	1623.0 (19.1)	1222.4 (12.5)	1.660 (1.531,1.799)	<0.0001
	≥65	1064.9 (19.6)	559.1 (11.8)	1.826 (1.635,2.040)	<0.0001
Hepatitis B Vaccine Series Completion (≥3 Doses)	18–34	866.9 (42.0)	771.6 (28.8)	1.796 (1.592,2.027)	<0.0001
	35–44	1479.3 (34.0)	732.2 (25.7)	1.486 (1.338,1.651)	<0.0001
	45–54	1310.6 (18.6)	796.2 (13.7)	1.440 (1.308,1.585)	<0.0001
	55–64	1123.0 (13.2)	888.5 (9.1)	1.530 (1.394,1.679)	<0.0001
	≥65	735.4 (13.5)	391.9 (8.3)	1.740 (1.529,1.980)	<0.0001
Vaccine-Mediated Immunity	18–34	1225.7 (59.4)	1352.2 (50.4)	1.442 (1.284,1.619)	<0.0001
	35–44	2336.8 (53.7)	1053.5 (37.0)	1.972 (1.791,2.173)	<0.0001
	45–54	3050.3 (43.3)	1515.7 (26.1)	2.164 (2.008,2.333)	<0.0001
	55–64	2868.6 (33.8)	2544.2 (26.0)	1.457 (1.367,1.552)	< 0.0001
	≥65	1847.4 (34.0)	1335.9 (28.2)	1.316 (1.209,1.432)	<0.0001

Outcome evets (%) are reported.

Table S4. Association between HBVIDV program and outcomes in original sample, stratified by living area

**Outcomes**	**Living area**	**Treatment group (n=6160)**	**Control group (n=200020)**	**Unadjusted OR (95% CI)**	**Adjusted OR (95% CI)** [a]	***p* Value**
Hepatitis B Vaccination (≥1 Dose)	Urban	333 (20.1)	1949 (20.5)	0.971 (0.853,1.106)	1.160 (1.004,1.340)	0.0442
	Rural	1375 (30.6)	1830 (17.4)	2.092 (1.929,2.269)	2.516 (2.298,2.756)	<0.0001
Hepatitis B Vaccine Series Completion (≥3 Doses)	Urban	253 (15.2)	1456 (15.3)	0.992 (0.858,1.147)	1.210 (1.031,1.419)	0.0195
	Rural	1047 (23.3)	1383 (13.1)	2.006 (1.834,2.193)	2.330 (2.110,2.574)	<0.0001
Vaccine-Mediated Immunity	Urban	609 (36.7)	3149 (33.2)	1.167 (1.047,1.300)	1.440 (1.283,1.617)	<0.0001
	Rural	1730 (38.4)	3061 (29.1)	1.524 (1.416,1.640)	1.770 (1.632,1.919)	<0.0001

Outcome evets (%) are reported.

[a]Model was adjusted for age, gender, educational level, employment condition, economic level, self-rated socioeconomic status, multimorbidity, self-rated health condition.

Table S5. Association between HBVIDV program and outcomes in propensity score-matched sample, stratified by living area

**Outcomes**	**Living area**	**Treatment group (n=5793)**	**Control group (n=5793)**	**OR (95% CI)**	***p* Value**
Hepatitis B Vaccination (≥1 Dose)	Urban	333 (20.1)	323 (19.5)	1.034 (0.872,1.227)	0.7009
	Rural	1348 (32.6)	710 (17.2)	2.338 (2.107,2.593)	<0.0001
Hepatitis B Vaccine Series Completion (≥3 Doses)	Urban	253 (15.2)	232 (14.0)	1.102 (0.909,1.337)	0.3228
	Rural	1025 (24.8)	529 (12.8)	2.251 (2.005,2.526)	<0.0001
Vaccine-Mediated Immunity	Urban	609 (36.7)	502 (30.4)	1.330 (1.151,1.537)	0.0001
	Rural	1602 (38.8)	1094 (26.4)	1.762 (1.605,1.934)	<0.0001

Outcome evets (%) are reported.

Table S6. Association between HBVIDV program and outcomes in inverse probability of treatment-weighted sample, stratified by living area

**Outcomes**	**Living area**	**Treatment group (n=6160)**	**Control group (n=20020)**	**OR (95% CI)**	***p* Value**
Hepatitis B Vaccination (≥1 Dose)	Urban	2387.6 (19.9)	2273.8 (20.4)	0.964 (0.904,1.028)	0.2618
	Rural	4840.0 (31.5)	2507.8 (17.0)	2.251 (2.131,2.378)	<0.0001
Hepatitis B Vaccine Series Completion (≥3 Doses)	Urban	1820.7 (15.1)	1687.9 (15.2)	0.997 (0.928,1.071)	0.9362
	Rural	3694.5 (24.1)	1892.5 (12.8)	2.156 (2.029,2.291)	<0.0001
Vaccine-Mediated Immunity	Urban	5119.6 (42.6)	3632.6 (32.7)	1.528 (1.448,1.612)	<0.0001
	Rural	6209.1 (40.5)	4168.9 (28.2)	1.727 (1.645,1.812)	<0.0001

Outcome evets (%) are reported.
