# Supplementary material for: Effect of a Community-Based Hepatitis B Virus Infection Detection Combined with Vaccination Program in China
Source: Vaccines (Basel). 2021 Dec 24;10(1):19. doi: 10.3390/vaccines10010019 (PMC8777927; doi:10.3390/vaccines10010019)

File S2 Sensitivity analysis of the association between HBVIDV program and serological evidence of vaccine-mediated immunity

Table S1. Characteristic of total sample, original sample, propensity score-matched sample, and inverse probability of treatment-weighted sample

Table S2. Association between HBVIDV program and vaccine-mediated immunity

Table S3. Association between HBVIDV program and vaccine-mediated immunity in original sample, stratified by age and living area

Table S4. Association between HBVIDV program and vaccine-mediated immunity in propensity score-matched sample, stratified by age and living area

Table S5. Association between HBVIDV program and vaccine-mediated immunity in Inverse probability of treatment-weighted sample, stratified by age and living area

Figure S1. Flowchart of study participant selection for HBVIDV program from July 2019 to June 2020.

Table S1. Characteristic of total sample, original sample, propensity score-matched sample, and inverse probability of treatment-weighted sample

Characteristic	Total sample (N=48063)	Original sample			Propensity 1:1 Matching			IPTW sample		
		Treatment group (N=11540)	Control group (N=36523)	SMD	Treatment group (N=11063)	Control group (N=11063)	SMD	Treatment group (N=11540)	Control group (N=36523)	SMD
Age				0.362			0.025			0.003
18–34	5712 (11.9)	551 (4.8)	5161 (14.1)		551 (5.0)	599 (5.4)		4462.5 (9.1)	5885.3 (12.3)	
35–44	6032 (12.6)	1283 (11.1)	4749 (13.0)		1283 (11.6)	1259 (11.4)		7926.4 (16.2)	5761.3 (12.1)	
45–54	11075 (23.0)	2509 (21.7)	8566 (23.5)		2509 (22.7)	2531 (22.9)		12309.5 (25.2)	11034.6 (23.1)	
55–64	16792 (34.9)	4345 (37.7)	12447 (34.1)		4171 (37.7)	4311 (39.0)		15782.7 (32.2)	16988.7 (35.6)	
≥65	8452 (17.6)	2852 (24.7)	5600 (15.3)		2549 (23.0)	2353 (21.4)		8466.1 (17.3)	8088.3 (16.9)	
Gender, male	18553 (38.6)	4131 (35.8)	14422 (39.5)	0.076	4002 (36.2)	3933 (35.6)	0.013	18543.8 (37.9)	184119.4 (38.6)	0.014
Education level				0.221			0.042			0.050
Illiterate	1007 (2.1)	252 (2.2)	755 (2.1)		252 (2.3)	262 (2.4)		846.1 (1.7)	1065.7 (2.2)	
Primary school/Junior high school	17415 (36.2)	5172 (44.8)	12243 (33.5)		5172 (46.8)	5067 (45.8)		17339.6 (35.4)	17265.2 (36.2)	
Senior high school	11397 (23.7)	2580 (15.9)	8817 (24.1)		2580 (23.3)	2764 (25.0)		11075.6 (22.6)	11651.4 (24.4)	
College graduate or above	8619 (17.9)	1839 (15.9)	6780 (18.6)		1839 (16.6)	1940 (17.5)		9445.9 (19.3)	8591.2 (18.0)	
Missing	9625 (20.0)	1697 (14.7)	7928 (21.7)		1220 (11.0)	1030 (9.3)		10240.0 (20.9)	9184.6 (19.2)	
Employment condition				0.290			0.025			0.056
Working	19939 (40.2)	3803 (33.0)	15536 (42.5)		3803 (34.4)	4240 (38.3)		15143.4 (30.9)	20490.0 (42.9)	
Retired	16580 (34.5)	5765 (50.0)	10815 (29.6)		5288 (47.8)	5014 (45.3)		19233.7 (39.3)	15280.4 (32.0)	
Unemployed	3108 (6.5)	930 (8.1)	2178 (6.0)		930 (8.4)	773 (7.0)		5068.3 (10.4)	2732.7 (5.7)	
Student	78 (0.2)	12 (0.1)	66 (0.2)		12 (0.1)	6 (0.1)		94.3 (0.2)	70.6 (0.2)	
Missing	8958 (18.6)	1030 (8.9)	7928 (21.7)		1030 (9.3)	1030 (9.3)		9407.6 (19.2)	9184.6 (19.2)	

Continued

Table S1 Continued

Characteristic	Total sample (N=48063)	Original sample			Propensity 1:1 Matching			IPTW sample		
		Treatment group (N=11540)	Control group (N=36523)	SMD	Treatment group (N=11063)	Control group (N=11063)	SMD	Treatment group (N=11540)	Control group (N=36523)	SMD
Economic level				0.179			0.055			0.044
Far below average	2261 (4.7)	511 (4.4)	1750 (4.8)		511 (4.6)	574 (5.2)		1969.8 (4.0)	2388.6 (5.0)	
Below average	7175 (14.9)	1999 (17.3)	5176 (14.2)		1999 (18.1)	1874 (16.9)		7978.7 (15.7)	7032.7 (14.7)	
Average	27911 (58.1)	7091 (61.5)	20820 (57.0)		7091 (64.1)	7359 (66.5)		28061 (57.3)	28047.7 (58.7)	
Above average	974 (2.0)	208 (1.8)	766 (2.1)		208 (1.9)	208 (1.9)		851.9 (1.7)	995.2 (2.1)	
Far above average	119 (0.3)	30 (0.3)	89 (0.2)		30 (0.3)	20 (0.2)		103.7 (0.2)	116.2 (0.2)	
Missing	9623 (20.0)	1701 (14.7)	7922 (21.7)		1224 (11.1)	1025 (9.3)		10282.1 (21.0)	9177.9 (19.2)	
Self-rated socioeconomic status				0.177			0.059			0.047
Higher	1006 (2.1)	244 (2.1)	762 (2.1)		244 (2.2)	250 (2.3)		967.4 (2.0)	1023.6 (2.1)	
Equality	30056 (62.5)	7708 (66.8)	22348 (61.2)		7708 (69.7)	7905 (71.5)		30024.5 (61.3)	30157.5 (63.2)	
Lower	7346 (15.3)	1877 (16.3)	5469 (15.0)		1877 (17.0)	1870 (16.9)		7628.0 (15.6)	7363.4 (15.4)	
Missing	9655 (20.1)	1711 (14.8)	7944 (21.8)		1234 (11.2)	1038 (9.4)		10327.3 (21.1)	9213.7 (19.3)	
Living area				0.496			0.004			0.023
Urban	19897 (41.4)	2759 (23.9)	17138 (46.9)		2759 (24.9)	2740 (24.8)		19794.4 (40.4)	19858.2 (41.6)	
Rural	28166 (58.6)	8781 (76.1)	19385 (53.1)		8304 (75.1)	8323 (75.2)		29152.8 (59.6)	27900 (58.4)	
Multimorbidity				0.154			0.058			0.047
Yes	8177 (17.0)	2881 (25.0)	5296 (14.5)		6955 (62.9)	7136 (64.5)		8721.2 (17.8)	8175.9 (17.1)	
No	30248 (62.9)	6955 (60.3)	23293 (63.8)		2881 (26.0)	2891 (26.1)		29927.0 (61.1)	30379.9 (63.6)	
Missing	9638 (20.1)	1704 (14.8)	7934 (21.7)		1227 (11.1)	1036 (9.4)		10299.1 (21.0)	9202.4 (19.3)	

Continued

Table S1 Continued

Characteristic	Total sample (N=48063)	Original sample			Propensity 1:1 Matching			IPTW sample		
		Treatment group (N=11540)	Control group (N=36523)	SMD	Treatment group (N=11063)	Control group (N=11063)	SMD	Treatment group (N=11540)	Control group (N=36523)	SMD
Self-rated health condition				0.203			0.057			0.038
Very unhealthy	1001 (2.1)	241 (2.1)	760 (2.1)		241 (2.2)	272 (2.5)		813.2 (1.7)	1070.0 (2.2)	
Unhealthy	2637 (5.5)	726 (6.3)	1911 (5.2)		726 (6.6)	719 (6.5)		2693.6 (5.5)	2637.5 (5.5)	
Fair healthy	12390 (25.8)	3640 (31.5)	8750 (24.0)		3640 (32.9)	3727 (33.7)		13507.7 (27.6)	12258.9 (25.7)	
Healthy	17858 (37.2)	4226 (36.6)	13623 (37.3)		4226 (38.2)	4345 (39.3)		17048.1 (34.8)	18143.6 (38.0)	
Very healthy	4522 (9.4)	996 (8.6)	3526 (9.7)		996 (9.0)	962 (8.7)		4557.9 (9.3)	4434.4 (9.3)	
Missing	9655 (20.1)	1711 (14.8)	7944 (21.8)		1234 (11.2)	1038 (9.4)		10327.3 (21.1)	9213.7 (19.3)	

Abbreviations: SMD, standard mean difference; IPTW, inverse probability of treatment weighting;

Categorical variables are reported as number (%).

Table S2. Association between HBVIDV program and vaccine-mediated immunity

	**Treatment group**	**Control group**	**OR (95% CI)**	***p* Value**
Original sample unadjusted model	4298 (37.2)	11155 (30.5)	1.350 (1.292,1.410)	<0.0001
Original sample adjusted model[a]	4298 (37.2)	11155 (30.5)	1.579 (1.506,1.656)	<0.0001
Propensity 1:1 Matching	4117 (37.2)	3046 (27.5)	1.560 (1.474,1.651)	<0.0001
IPTW sample	19141.5 (39.1)	14224.1 (29.8)	1.514 (1.474,1.555)	<0.0001

Abbreviations: OR (95% CI), Odds Ratio (95% Confidence Interval). IPTW, inverse probability of treatment weighting.

Outcome events (%) are reported.

[a]Model was adjusted for age, gender, educational level, employment condition, economic level, self-rated socioeconomic status, living area, multimorbidity, self-rated health condition.

Table S3. Association between HBVIDV program and vaccine-mediated immunity in original sample, stratified by age and living area

	Treatment group (N=11540)	Control group (N=36523)	Unadjusted OR (95% CI)	Adjusted OR (95% CI)	*p* value	*p* for interaction
Age[a]						<0.0001
18–34	318 (57.7)	2493 (48.3)	1.461 (1.223,1.745)	1.410 (1.170,1.700)	0.0003	
35–44	596 (46.5)	1656 (34.9)	1.620 (1.430,1.836)	1.650 (1.446,1.882)	<0.0001	
45–54	902 (36.0)	2170 (25.3)	1.655 (1.505,1.819)	1.778 (1.610,1.963)	<0.0001	
55–64	1512 (34.8)	3234 (26.0)	1.521 (1.421,1.638)	1.566 (1.447,1.695)	<0.0001	
≥65	970 (34.0)	1602 (28.6)	1.286 (1.168,1.417)	1.358 (1.217,1.515)	<0.0001	
Living area[b]						<0.0001
Urban	1010 (36.6)	5596 (32.7)	1.191 (1.095,1.295)	1.349 (1.235,1.472)	<0.0001	
Rural	3288 (37.4)	5559 (28.7)	1.489 (1.412,1.570)	1.668 (1.576,1.765)	<0.0001	

Outcome events (%) are reported.

[a]Model was adjusted for gender, educational level, employment condition, economic level, self-rated socioeconomic status, living area, multimorbidity, self-rated health condition.

[b]Model was adjusted for age, gender, educational level, employment condition, economic level, self-rated socioeconomic status, multimorbidity, self-rated health condition.

Table S4. Association between HBVIDV program and vaccine-mediated immunity in propensity score-matched sample, stratified by age and living area

	Treatment group (n=11063)	Control group (n=11063)	OR (95% CI)	*p* value	*p* for interaction
Age					<0.0001
18–34	318 (57.7)	299 (49.9)	1.369 (1.085,1.728)	0.0081	
35–44	596 (46.5)	429 (34.1)	1.678 (1.430,1.970)	<0.0001	
45–54	902 (36.0)	601 (23.8)	1.802 (1.594,2.037)	<0.0001	
55–64	1440 (34.5)	1093 (25.4)	1.552 (1.414,1.705)	<0.0001	
≥65	861 (33.8)	624 (26.4)	1.421 (1.257,1.607)	<0.0001	
Living area					<0.0001
Urban	1010 (36.6)	840 (30.7)	1.306 (1.168,1.461)	<0.0001	
Rural	3107 (37.4)	2206 (26.5)	1.658 (1.552,1.771)	<0.0001	

Outcome events (%) are reported.

Table S5. Association between HBVIDV Program and vaccine-mediated immunity in inverse probability of treatment-weighted sample, stratified by age and living area

	Treatment group (n=11540)	Control group (n=36523)	OR (95% CI)	*p* value	*p* for interaction
Age					<0.0001
18–34	2558.4 (57.3)	2839.5 (48.3)	1.441 (1.333,1.559)	<0.0001	
35–44	3806.1 (48.0)	1995.9 (34.6)	1.743 (1.625,1.869)	<0.0001	
45–54	4624.9 (37.6)	2757.2 (25.0)	1.807 (1.707,1.912)	<0.0001	
55–64	5302.8 (33.6)	4357.2 (25.7)	1.467 (1.399,1.539)	<0.0001	
≥65	2849.4 (33.7)	2274.4 (28.1)	1.297 (1.214,1.386)	<0.0001	
Living area					<0.0001
Urban	7870.6 (39.8)	6430.1 (32.4)	1.378 (1.323,1.436)	<0.0001	
Rural	11270.1 (38.7)	7794.1 (27.9)	1.626 (1.570,1.684)	<0.0001	

Outcome events (%) are reported.

Figure S1. Flowchart of study participant selection for HBVIDV program from July 2019 to June 2020.

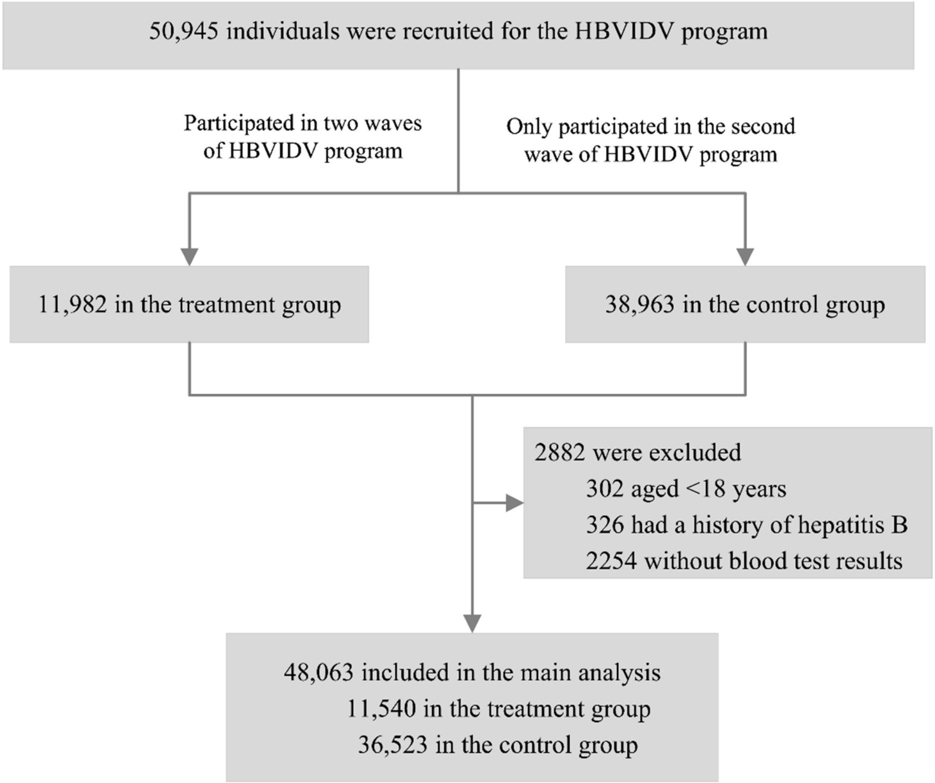

Supplement: Supplementary file 1 [file vaccines-10-00019-s001.zip › File S2.pdf]
