# Supplementary material for: Effect of a Community-Based Hepatitis B Virus Infection Detection Combined with Vaccination Program in China
Source: Vaccines (Basel). 2021 Dec 24;10(1):19. doi: 10.3390/vaccines10010019 (PMC8777927; doi:10.3390/vaccines10010019)
Supplement: Supplementary file 1 [file vaccines-10-00019-s001.zip › File S3.pdf]

File S3 Sensitivity analysis of the association between HBVIDV program and serological evidence of vaccine-mediated immunity in complete data set

Table S1. Proportion of missing data

Table S2. Characteristic of total sample, original sample, propensity score-matched sample, and inverse probability of treatment-weighted sample

Table S3. Association between HBVIDV program and vaccine-mediated immunity

Table S4. Association between HBVIDV program and vaccine-mediated immunity in original sample, stratified by age and living area

Table S5. Association between HBVIDV program and vaccine-mediated immunity in propensity score-matched sample, stratified by age and living area

Table S6. Association between HBVIDV program and vaccine-mediated immunity in inverse probability of treatment-weighted sample, stratified by age and living area

Table S1. Proportion of missing data

Variables	n	%
Education level	9625	20
Employment condition	8958	18.6
Economic level	9623	20
Self-rated socioeconomic status	9655	20
Multimorbidity	9638	20
Self-rated health condition	9655	20

%=n/N. N was the number the number after excluding younger than 18 years old, past history of hepatitis B, and lack of blood test results, which is 48063.

Table S2. Characteristic of total sample, original sample, propensity score-matched sample, and inverse probability of treatment-weighted sample

Characteristic	Total sample (N=38180)	Original sample			Propensity 1:1 Matching			IPTW sample		
		Treatment group (N=9621)	Control group (N=28559)	SMD	Treatment group (N=9621)	Control group (N=9621)	SMD	Treatment group (N=9621)	Control group (N=28559)	SMD
Age group				0.373			0.035			0.032
18–34	4653 (12.2)	483 (5.02)	4170 (14.6)		483 (5.02)	534 (5.6)		3448.6 (9.2)	4675.1 (12.2)	
35–44	4656 (12.2)	1045 (10.9)	3611 (12.6)		1045 (10.9)	1014 (10.5)		5857.1 (15.7)	4371.3 (11.4)	
45–54	8642 (22.6)	2050 (21.3)	6592 (23.1)		2050 (21.3)	2036 (21.2)		8639.1 (23.1)	8567.5 (22.4)	
55–64	13691 (35.9)	3656 (38.0)	10035 (35.1)		3656 (38.0)	3941 (41.0)		11777.6 (31.6)	14447.2 (37.8)	
≥65	6538 (17.1)	2387 (24.8)	4151 (14.5)		2387 (24.8)	2096 (21.8)		7607.9 (20.4)	6170.2 (16.1)	
Gender, male	14916 (39.1)	3523 (36.6)	11393 (39.9)	0.067	3523 (36.6)	3375 (35.1)	0.032	13464.2 (36.1)	14834.6 (38.8)	
Education level							0.052			0.057
Illiterate	1001 (2.6)	246 (2.6)	755 (2.6)		246 (2.6)	223 (2.3)		912.3 (2.4)	999.7 (2.6)	0.040
Primary school/Junior high school	17275 (45.3)	5045 (52.4)	12230 (42.8)		5045 (52.4)	4747 (49.3)		16759.3 (44.9)	17073.1 (44.7)	
Senior high school	11323 (29.7)	2516 (26.2)	8807 (30.8)		2516 (26.2)	2772 (28.8)		10155.2 (27.2)	11677.4 (30.5)	
College graduate or above	8581 (22.5)	1814 (18.9)	6767 (23.7)		1814 (18.9)	1879 (19.5)		9503.6 (25.5)	8481.1 (22.2)	
Employment condition				0.264			0.010			0.042
Working	19262 (50.5)	3747 (39.0)	15515 (54.3)		3747 (39.0)	3764 (39.1)		18088.5 (48.5)	19156.6 (50.1)	
Retired	15764 (41.3)	4959 (51.5)	10805 (37.8)		4959 (51.5)	5005 (52.0)		15773.8 (42.3)	15980.4 (41.8)	
Unemployed	3082 (8.1)	909 (9.5)	2173 (7.6)		909 (9.5)	837 (8.7)		3437.0 (9.2)	3018.6 (7.9)	
Student	72 (0.2)	6 (0.1)	66 (0.2)		6 (0.1)	15 (0.2)		31.0 (0.1)	75.8 (0.2)	

Continued

Table S2 Continued

Characteristic	Total sample	Original sample			Propensity 1:1 Matching			IPTW sample		
	(N=38180)	Treatment group (N=9621)	Control group (N=28559)	SMD	Treatment group (N=9621)	Control group (N=9621)	SMD	Treatment group (N=9621)	Control group (N=28559)	SMD
Economic level				0.010			0.017			0.001
Far below average	2240 (5.9)	492 (5.1)	1748 (6.1)		492 (5.1)	553 (5.8)		1972.9 (5.3)	2346.1 (6.1)	
Below average	7109 (18.6)	1944 (20.2)	5165 (18.1)		1944 (20.2)	1715 (17.8)		7334.0 (19.7)	6918.4 (18.1)	
Average	27741 (72.7)	6948 (72.2)	20793 (72.8)		6948 (72.2)	7125 (74.1)		27017.6 (72.4)	27866.5 (72.9)	
Above average	972 (2.6)	207 (2.2)	765 (2.7)		207 (2.2)	198 (2.1)		908.0 (2.4)	984.7 (2.6)	
Far above average	118 (0.3)	30 (0.3)	88 (0.3)		30 (0.3)	30 (0.3)		97.8 (0.3)	115.9 (0.3)	
Self-rated socioeconomic status				0.002			0.007			0.001
Higher	1000 (2.6)	240 (2.5)	760 (2.7)		240 (2.5)	211 (2.2)		1078.3 (2.9)	991.7 (2.6)	
Equality	29896 (78.3)	7564 (78.6)	22332 (78.2)		7564 (78.6)	7649 (79.5)		29062.2 (77.9)	29970.7 (78.4)	
Lower	7284 (19.1)	1817 (18.9)	5467 (19.1)		1817 (18.9)	1761 (18.3)		7189.8 (19.3)	7269.0 (19.0)	
Living area				0.587			0.009			0.026
Urban	17978 (47.1)	2543 (26.4)	15435 (54.1)		2543 (26.4)	2507 (26.1)		17039.8 (45.7)	17955.8 (47.0)	
Rural	20202 (52.9)	7078 (73.6)	13124 (46.0)		7078 (73.6)	7114 (73.9)		20290.5 (54.4)	20275.6 (53.0)	
Multimorbidity	8033 (21.0)	2740 (28.5)	5293 (18.5)	0.236	2740 (28.5)	2671 (27.8)	0.016	8377.6 (22.4)	8090.2 (21.2)	0.031
Self-rated health condition				0.098			0.003			0.026
Very unhealthy	986 (2.6)	228 (2.4)	758 (2.7)		228 (2.4)	230 (2.4)		860.3 (2.3)	1019.3 (2.7)	
Unhealthy	2605 (6.8)	695 (7.2)	1910 (6.7)		695 (7.2)	651 (6.8)		2663.3 (7.1)	2578.2 (6.7)	
Fair healthy	122290 (32.2)	3545 (36.9)	8745 (30.6)		3545 (36.9)	3492 (36.3)		12929.1 (34.6)	12209.3 (31.9)	
Healthy	17786 (46.6)	4165 (43.3)	13621 (47.7)		4165 (43.3)	4373 (45.5)		16521.4 (44.3)	18024.7 (47.2)	
Very healthy	4513 (11.8)	988 (10.3)	3525 (12.3)		988 (10.3)	875 (9.1)		4356.2 (11.7)	4399.8 (11.5)	

Abbreviations: SMD, standard mean difference; IPTW, inverse probability of treatment weighting. Categorical variables are reported as number (%).

Table S3. Association between HBVIDV program and vaccine-mediated immunity

	Treatment group	Control group	OR (95% CI)	*p* Value
Original sample unadjusted model	3537 (36.7)	8503 (29.8)	1.371 (1.306,1.440)	<0.0001
Original sample adjusted model[a]	3537 (36.7)	8503 (29.8)	1.651 (1.567,1.741)	<0.0001
Propensity 1:1 Matching	3537 (36.8)	2553 (26.5)	1.609 (1.514,1.711)	<0.0001
IPTW sample	14711.1 (39.1)	11029.4 (28.7)	1.599 (1.551,1.648)	<0.0001

Abbreviations: OR (95% CI), Odds Ratio (95% Confidence Interval). IPTW, inverse probability of treatment weighting.

Outcome events (%) are reported.

[a]Model was adjusted for age, gender, educational level, employment condition, economic level, self-rated socioeconomic status, living area, multimorbidity, self-rated health condition.

Table S4. Association between HBVIDV program and vaccine-mediated immunity in original sample, stratified by age and living area

	Treatment group (N=9621)	Control group (N=28559)	Unadjusted OR (95% CI)	Adjusted OR (95% CI)	*p* Value	*p* for interaction
Age[a]						<0.0001
18–34	289 (59.8)	2002 (48.0)	1.613 (1.332,1.954)	1.404 (1.140,1.729)	0.0014	
35–44	492 (47.1）	1228 (34.0)	1.727 (1.502,1.985)	1.726 (1.484,2.007)	<0.0001	
45–54	712 (34.7)	1583 (24.0)	1.684 (1.513,1.874)	1.850 (1.649,2.076)	<0.0001	
55–64	1239 (33.9)	2531 (25.2)	1.520 (1.400,1.650)	1.660 (1.522,1.810)	<0.0001	
≥65	805 (33.7)	1159 (27.9)	1.314 (1.178,1.464)	1.427 (1.274,1.598)	<0.0001	
Living area[b]						<0.0001
Urban	918 (36.1)	4936 (32.0)	1.202 (1.101,1.312)	1.412 (1.287,1.549)	<0.0001	
Rural	2619 (37.0)	3567 (27.2)	1.574 (1.480,1.674)	1.755 (1.645,1.872)	<0.0001	

Outcome events (%) are reported.

[a]Model was adjusted for gender, educational level, employment condition, economic level, self-rated socioeconomic status, living area, multimorbidity, self-rated health condition.

[b]Model was adjusted for age, gender, educational level, employment condition, economic level, self-rated socioeconomic status, multimorbidity, self-rated health condition.

Table S5. Association between HBVIDV program and vaccine-mediated immunity in propensity score-matched sample, stratified by age and living area

	**Treatment group (n=9621)**	**Control group (n=9621)**	**OR (95% CI)**	***p* Value**	***p* for interaction**
Age					<0.0001
18–34	289 (59.8)	281 (52.6)	1.341 (1.046,1.720)	0.0208	
35–44	492 (47.1)	348 (34.3)	1.703 (1.426,2.034)	<0.0001	
45–54	712 (34.7)	463 (22.7)	1.808 (1.575,2.075)	<0.0001	
55–64	1239 (33.9)	931 (23.6)	1.657 (1.499,1.832)	<0.0001	
≥65	805 (33.7)	530 (25.3)	1.503 (1.320,1.712)	<0.0001	
Living area					<0.0001
Urban	918 (36.1)	735 (29.3)	1.362 (1.210,1.533)	<0.0001	
Rural	2619 (37.0)	1818 (25.6)	1.711 (1.592,1.838)	<0.0001	

Outcome events (%) are reported.

Table S6. Association between HBVIDV program and vaccine-mediated immunity in inverse probability of treatment-weighted sample, stratified by age and living area

	**Treatment group (N=9621)**	**Control group (N=28559)**	**OR (95% CI)**	***p* Value**	***p* for interaction**
Age					<0.0001
18–34	2108.0 (61.1)	2254.8 (48.2)	1.688 (1.544,1.845)	<0.0001	
35–44	2943.0 (50.3)	1487.9 (34.0)	1.957 (1.805,2.122)	<0.0001	
45–54	3118.0 (36.1)	2025.3 (23.6)	1.824 (1.707,1.949)	<0.0001	
55–64	3942.0 (33.5)	3568.2 (24.7)	1.534 (1.453,1.619)	<0.0001	
≥65	2565.3 (33.7)	1670.5 (27.1)	1.370 (1.273,1.475)	<0.0001	
Living area					<0.0001
Urban	6906.4 (40.5)	5658.2 (31.5)	1.481 (1.418,1.548)	<0.0001	
Rural	7769.8 (38.3)	5348.5 (26.4)	1.732 (1.660,1.806)	<0.0001	

Outcome events (%) are reported
